# Effects of Adding Single Joint Exercises to a Resistance Training Programme in Trained Women

**DOI:** 10.3390/sports6040160

**Published:** 2018-11-28

**Authors:** Matheus Barbalho, Victor Silveira Coswig, Rodolfo Raiol, James Steele, James Fisher, Antonio Paoli, Paulo Gentil

**Affiliations:** 1 Faculdade de Educação Física e Dança, Universidade Federal de Goiás, Goiânia 74690-900, Brazil; paulogentil@hotmail.com; 2 Faculdade de Educação Física, Universidade Federal do Pará, Castanhal 68746-630, Brazil; vcoswig@gmail.com; 3 Centro de Ciências Biológicas e da Saúde, Centro Universitário do Pará, Belém 66040-020, Brazil; rodolforaiol@gmail.com; 4 Ukactive Research Institute, London WC1R 4HE, UK; james.steele@solent.ac.uk; 5 School of Sport, Health, and Social Science, Southampton Solent University, Southampton SO14 0AA, UK; james.fisher@solent.ac.uk; 6 Department of Biomedical Sciences, University of Padua, 35100 Padua, Italy; antonio.paoli@unipd.it

**Keywords:** strength training, muscle hypertrophy, training volume, exercise selection, isolation exercise

## Abstract

Background: The present study’s aim was to compare the changes in muscle performance and anthropometric measures in trained women performing RT programs composed only of MJ exercises or programmes that involve the addition of SJ exercises. Methods: Seventeen trained women were randomised to MJ or MJ+SJ. Both groups performed the same MJ exercises following a nonlinear periodisation model for 8 weeks. The only difference was that the MJ+SJ group also performed SJ exercises. The participants were tested for 10 repetition maximum (10 RM), flexed arm circumference, and both biceps and triceps skinfold. Results: Both groups significantly increased 10 RM load for the bench press (12.6% MJ and 9.2% MJ+SJ), triceps (15.6% MJ and 17.9% MJ+SJ), pull down (9.8% MJ and 8.3% MJ+SJ), biceps (14.0% MJ and 13.0% MJ+SJ), leg press (15.2% MJ and 12.8% MJ+SJ) and knee extension (10.2% MJ and 9.1% MJ+SJ). The decreases in triceps (−5.1% MJ and −5.3% MJ+SJ) and biceps (−6.5% MJ and −5.7% MJ+SJ) skinfolds were also significant as were the increases in arm circumference (1.47% MJ and 1.58% MJ+SJ). In all tests there was nothing significantly different between groups. Conclusions: The use of SJ exercises as a complement to a RT programme containing MJ exercises brings no additional benefit to trained women.

## 1. Introduction

Resistance training (RT) can bring many benefits to women, such as increases in muscle strength [1] and bone mineral density [2], improvements in maternal health and perinatal outcomes during pregnancy [3], changes in body composition [4] and improvements in health-related outcomes in older age [5]. It is argued that optimisation of results produced from a RT programme are dependent on the manipulation of a number of variables including: Exercise order, rest interval, number of exercise’s performed and exercise selection [6]. RT exercises are often selected based on the muscles involved in the movement and it is usually believed that some exercises offer greater potential to develop a given muscle group compared with others [6]. The primary distinction made when considering RT exercises is usually whether they are multi (MJ) or single joint (SJ), depending on the number of joints involved. Recent studies have reported no differences in muscle size and strength between SJ only or MJ only exercises upon upper arm adaptations [7] and there is also evidence that the addition of SJ exercises to a MJ exercise RT programme does not increase the gains in muscle size and strength in untrained [8] or trained men [9]. Based on this, the sole use of MJ exercises (due to the fact that they target several muscle groups simultaneously) has been suggested to be an interesting option due to its time efficiency [10], especially if we consider that lack of time is a common barrier to exercise adherence [11]. Indeed, it has recently been suggested that a simple MJ exercise based RT intervention should be considered as the minimal effective dose prophylactic for age related functional decline [12]. However, recent studies showed that the addition of SJ exercises to an MJ RT programme lead to higher increases in flexed arm circumference in untrained women and men, whilst no differences were found for biceps and triceps skinfolds of muscle strength [13].

Whilst evidence suggests the lack of necessity for most SJ exercises, literature regarding the topic has thus far been limited to male participants in long-term adaptations [10], with the exception of Barbalho et al. [13]. Although there is evidence that men and women trained respond similarly to RT programmes [1], other studies show different muscle size and strength gains between sexes [14]. Moreover, there are many studies reporting that men and women have different acute responses to RT, especially regarding fatigability [15,16,17,18], muscle recovery [19] and muscle activation [20]. Another important gap in the current literature is that long term studies have typically been limited to upper body muscles (usually arm muscles) since upper and lower body muscles often have different responses to RT, and the lower limbs are supposed to require additional volume and exercises [21], it is unclear whether or not, despite having a lack of additional benefit for upper body musculature, there may be benefit to adding SJ exercises to and RT programme for lower body musculature.

Our hypothesis is that resistance training with MJ exercises and with addition of SJ exercises, promote the same gains in muscular performance and anthropometric measures. Therefore, the purpose of the present study was to compare the changes in muscle performance and anthropometric measures in trained women performing a RT programme composed of only MJ and with the addition of SJ exercises. 

## 2. Materials and Methods

### 2.1. Experiment Overview

In order to examine the effects of performing SJ exercises on upper and lower body muscle strength and anthropometry, 20 young women with at least 1 year of previous RT experience were randomly divided into 2 groups. One group performed a RT programme containing only MJ exercises (MJ group), while the other added SJ to the same programme followed by the MJ group (MJ+SJ group). Training followed a nonlinear periodisation model for 8 weeks. Before and after the training period, the participants were tested for 10 repetition maximum (10 RM) in the bench press, triceps pulley, lat pulldown, biceps curl, leg press, and knee extension. Arm circumference, biceps and triceps skinfold were also measured to evaluate anthropometric changes. Training volume was not equated, because the difference was intended to be inherent to the protocols and to reflect the *addition* of SJ exercises to typical MJ exercise RT protocols.

### 2.2. 10-Repetition Maximum (10 RM)

Before and after the intervention, participants performed 10 RM tests in the bench press, elbow extension, lat pulldown, elbow flexion, leg press, and knee extension (Physicus, Pró; Auriflama, São Paulo, Brazil). Tests were divided in 3 consecutive days. In the first day, participants were tested for bench press and knee extension; the second involved lat pulldown and biceps; leg press and triceps were tested in the third day. We chose 10 RM instead of 1 RM because, when participants are training at high repetition ranges, it seems more suitable to evaluate performance through multiple repetition tests [22].

Participants warmed up with 10 repetitions at a comfortable self-selected load and then rested for 5 min. After the warm up the estimated 10 RM load was set based on the participants’ training history. If the volunteer was not able to perform 10 repetitions or performed more than 10 repetitions, the load was adjusted starting at 1 kg. Rest between attempts was set at 5 min and no more than three attempts were allowed in each session. The test-retest reliability coefficient (ICC) of this procedure was determined in our lab prior to conducting the study by performing two identical test sessions one week apart, the values ranged between 0.93 and 0.98. In that analysis the standard error of the measurement (SEM) was usually less than 3%.

### 2.3. Participants

**Figure 1:**
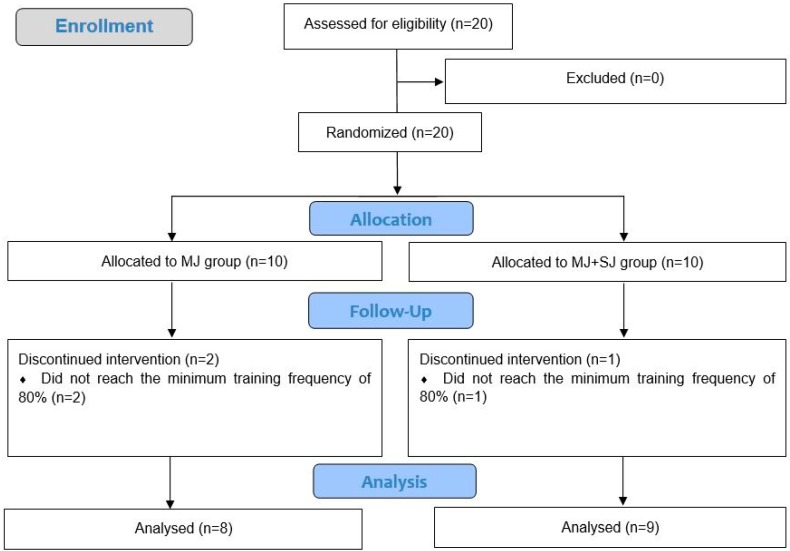
Flow diagram.

To participate in the study, volunteers had to be at least 18 years old and to not have any clinical conditions that would limit their participation or could be aggravated by the study protocol. Participants also had to have performed RT during the previous year at a frequency of at least 3 sessions per week, in which they used MJ and SJ exercises in their training routines. Minimum attendance for the studies training intervention was set at 80% based on previous findings [23], which led to the exclusion of three participants from the analysis (MJ *n* = 2, and MJ+SJ *n* = 1), as demonstrated in the flow diagram (Figure 1). Whilst there was no control of the participants’ diets, they were instructed to maintain their habitual diets and were intermittently questioned to check if any drastic changes occurred, such as the use of ergogenic aids and the adoption of different nutrient selection (i.e., increasing protein intake, decreasing carbohydrate intake, becoming vegetarian, etc.). After being informed of the experimental procedures, its risks and benefits, the participants signed an informed consent form. The study was approved by the local Ethics Committee under the number CAAE 69724617.7\.0000.5169. 

### 2.4. Anthropometric Measures

Flexed arm circumference (FAC), biceps and triceps skinfold were measured at the right side of the body in the week before the first training session, and 5 to 7 days after the last training session, due to muscle swelling. The participants were instructed to avoid RT for at least five days before the tests. For FAC, the arm was raised to a horizontal position in the sagittal plane, with the elbow at 90 degrees. The subject maximally contracted the elbow flexors, and the largest circumference was measured. Biceps and triceps skinfold were measured at the meso-humeral point while the arm was in the anatomical position hanging down the side of the body and relaxed. Three measures were taken and the average of the values was used during the analysis [24].

### 2.5. Training

Training was performed 6 times a week, divided in 3 different muscle groups, as shown in Table 1. Therefore, each muscle groups was trained twice a week with at least 72 h between sessions. All participants were supervised and monitored in all exercises, with a supervision ratio of at least 1 supervisor to 5 trainees [25]. 

Both groups performed the same MJ exercises, loads, repetition ranges, set endpoints (to momentary failure), and rest intervals, differing only in inclusion of SJ exercises for the MJ+SJ group. Specific exercises were included for plantar flexors and hamstrings to avoid muscle imbalances, since they are not highly involved in the MJ exercises used [10]. The protocol was based on a non-linear periodisation model because it has been suggested to produce better results in short-term studies [26,27]. During weeks 1 and 5, participants used loads permitting 12–15 repetitions before reaching momentary failure with 30–60 s of rest between sets. During weeks 2 and 6, loads permitting up to 4–6 repetitions before reaching momentary failure were used with 3–4 min of rest between sets. Weeks 3 and 7 involved loads permitting 10–12 repetitions before reaching momentary failure with 1–2 min of interval between sets. During weeks 4 and 8, participants used loads permitting 6–8 repetitions before reaching momentary failure with inter-set intervals of 2–3 min. Participants were instructed to perform every set up to momentary failure as previously defined by Steele et al. [28], and when they were able to perform more repetitions than suggested, the load was increased (1–5 kg) in alignment with the desired repetition range for the next training session. The volunteers were instructed to perform the concentric and eccentric phases in two seconds each, without pausing between contractions.

### 2.6. Statistical Analysis

All values are reported by means ± standard deviation. The independent variable was the group (MJ or MJ+SJ) and the dependant variables were the absolute change in the outcome variables (post- minus pre-test scores). Analysis of covariance (ANCOVA) was used to compare absolute change in each outcome variable between groups with pre-test scores used as a covariate. Further, 95% confidence intervals (CI) were examined for within group change. Significant within group change was considered to have occurred if the 95%CIs for changes did not cross zero. Statistical analysis was performed using JASP (version 0.8.1.2; University of Amsterdam, Amsterdam, The Netherlands), with alpha for significance accepted at ≤0.05.

## 3. Results

The characteristics of the participants are shown in Table 2. Table 3 presents the pre and post values for the variables analysed.

### 3.1. Muscle Performance Outcomes (10 RM)

Between groups comparisons using ANCOVA revealed no significant differences for changes in any muscle performance outcome. The 95%CIs also suggested that both groups significantly increased in the 10 RM load in the bench press (12.61% for MJ and 9.27% for MJ+SJ), elbow extension (15.64% for MJ and 17.94% for MJ+SJ), lat pull down (9.48% for MJ and 8.37% for MJ+SJ), elbow flexion (14.04% for MJ and 13.09% for MJ+SJ), leg press (15.26% for MJ and 12.85% for MJ+SJ) and knee extension (10.26% for MJ and 9.17% for MJ+SJ). Change for each muscle performance outcome in addition to the 95%CIs for the changes are shown in Table 4.

### 3.2. Anthropometric Outcomes (Biceps and Triceps Skinfolds, and FAC)

Between groups comparisons using ANCOVA revealed no significant differences for changes in any anthropometric outcome The 95%CIs also suggested that both groups significantly decreased in triceps (−5.14% for MJ and −5.30% for MJ+SJ) and biceps (−6.56% for MJ and −5.74% for MJ+SJ) skinfolds, and increased in arm circumference (1.47% for MJ and 1.58% for MJ+SJ). Change for each anthropometric outcome in addition to the 95%CIs for the changes are also shown in Table 4.

## 4. Discussion

The present study compared performance and anthropometric changes in trained women performing a RT programme composed of only MJ exercises compared with performing the same programme with the addition of SJ exercises. The results showed that both groups had significant improvements in all variables, but no difference was found between groups in these changes.

Our results are in agreement with what has previously been reported in trained [9] and untrained men [8]. Using a similar design, de França et al. [9] compared the changes in upper body muscle strength and size in trained men after 8 weeks performing RT with MJ+SJ exercises or MJ exercises only and found no difference in strength gains in the elbow flexors and extensors nor in anthropometric changes between groups. Interestingly, the increases reported in elbow flexion (4.99% and 6.42% for MJ and MJ+SJ, respectively), extension (10.60% vs. 9.79%, for MJ and MJ+SJ, respectively), and FAC (1.72% vs. 1.45%, for MJ and MJ+SJ, respectively) were similar to what we found in the present study. Although direct comparison cannot be made, this suggests a similar response in trained men and women, following the same trend previously reported in untrained subjects [1,29].

Our results seem to be in contrast with previous studies in untrained women [30] and men [13], where the addition of SJ exercises to an MJ RT programme lead to higher increases in flexed arm circumference. One possible explanation might be found in the study of Ogasawara et al. [31] that evaluated the time course of chest and triceps muscle hypertrophy in response to an MJ exercise (bench press). According to the results, triceps brachii shows a slower response, only becoming significant after 5 weeks of training, while chest muscles showed increases in the first week. These results suggested a slower hypertrophic response of the arm muscles. Based on this, the use of SJ exercises might provide benefits during this phase, at least for the triceps. However, after this initial period, muscle hypertrophy might get near the upper limit and no additional benefit is seem with the addition of SJ, which might explain the lack of results for trained individuals.

Despite supporting prior work comparing MJ only to MJ+SJ exercises in the upper body [9], the present study introduces novel findings as the lower body was also examined. As far as we know, this is the first study to compare the addition of SJ exercises to an MJ exercise programme in lower body performance in trained individuals. Previous studies suggested that quadriceps muscle activation during MJ exercises are similar to or even higher than during SJ exercises [10] and thus this may explain the lack of additional benefit. However, the addition of SJ exercise might be a way of increasing exercise volume for a particular muscle group and so still might be thought to offer benefit. This is perhaps due to the suggestion that the lower body may require additional volume to optimise adaptation [26]. However, the participants of the MJ group were likely already in the theoretical upper limit of the dose-response curve for both strength [32] and hypertrophy [33], which might explain the absence of benefits with the inclusion of SJ exercises. Another point of prominence is that, although the groups did not present any difference between them, the MJ group presented higher ES in the leg press than the MJ+SJ group.

It is important to note that isolated knee extension has been associated with knee pain in some cases [34] and its performance has been show to result in more unfavourable forces in the knee joint than MJ exercises [35,36]. In contrast, MJ exercises of the lower body show coactivation of the anterior and posterior musculature [37], which has, in turn, been shown to help stabilise the knee [38]. Therefore, avoiding SJ exercise might be an interesting strategy not only for long-term adherence due to time efficiency, but also for long-term safety. This may be particularly important to women that usually have higher rates of knee injury than men [39,40,41,42]. This is however something requiring further investigation to determine whether injury, degeneration, or pain prevalence differs between long term use of either MJ exercises only (leg press/squats) compared with SJ exercises only (knee extension). 

One important aspect of the present study was that the MJ group had similar increases to MJ+SJ group in single joint exercise, despite the fact that they did not perform this exercise in their routine. It is likely that the performance of SJ movements did not produce superior gains in performance because these exercises involve simple tasks that do not have a high degree of complexity with respect to the motor learning required to optimise them. In fact, neurological adaptations to SJ exercises may be largely attained primarily in the first few weeks of training [43]. Considering that our participants were trained and had a prior history of performing SJ exercises, the continued performance of specific SJ movements likely did not make further difference. However, studies in non-trained women would bring further insights into this topic.

Some limitations should be considered while interpreting our findings. First, it would be interesting to have an only-SJ group. However, in respect to focus in our main research question (addition of SJ exercises to a MJ programme) and considering logistic factors, we prefer to not include this group, which would be of interest in future research. Second, a nutrient intake control or monitoring would be relevant, especially for anthropometrics. Even with our request to subjects to maintain their regular diet, it still being a confusion factor and further research should add this monitoring by food logs, including using apps. 

## 5. Conclusions

Based on the present findings, we conclude that the use of SJ exercises as a complement to a RT programme containing MJ exercises brings no additional benefit to trained women in terms of muscle performance and anthropometry. In light of this, we suggest that the use of RT programmes containing only MJ exercises might be recommended with the purpose to provide a time efficient approach with no impairment in strength endurance or anthropometric measurements. 

## Figures and Tables

**Table 1:** Training programmes.

Monday and Thursdays	Tuesdays and Fridays	Wednesday and Saturdays
Barbell bench press	Lat pull down	45° leg press
Inclined barbell bench press	Neutral grip cable row	Barbell Back squat
Military press	Upright barbell row pronated	Seated knee flexion
Cable elbow extensions *	Barbell biceps curl *	Calf raises
-	-	Knee extension *

* Performed only by the MJ+SJ group.

**Table 2:** Characteristics of the participants (mean ± standard deviation).

	MJ (*n* = 8)	MJ+SJ (*n* = 9)
	Mean ± sd	Mean ± sd
Age (yrs)	25 ± 2.87	23.77 ± 3.92
Height (m)	165.37 ± 4.06	168.33 ± 3.24
Body Mass (kg)	61.12 ± 4.29	64.22 ± 4.38
Experience (yrs)	2.37 ± 0.74	2.55 ± 0.52

**Table 3:** Characteristics of the participant’s pre and post the training period (mean ± standard deviation).

	MJ Group				MJ+SJ Group			
	Pre	Post	*p*	ES	Pre	Post	*p*	ES
Bench press 10 RM (kg)	24.75 ± 4.03	27.87 ± 4.22	<0.001	0.35	26.33 ± 5.0	28.77 ± 5.04	<0.001	0.23
Triceps 10 RM (kg)	13.62 ± 3.20	15.75 ± 3.28	<0.001	0.31	13.66 ± 2.92	16.11 ± 3.41	<0.001	0.36
Pulldown 10 RM (kg)	25.0 ± 5.13	27.37 ± 4.87	<0.001	0.23	24.6 ± 3.74	26.66 ± 3.74	<0.001	0.26
Biceps 10 RM (kg)	14.25 ± 1.67	16.25 ± 1.67	<0.001	0.51	14.44 ± 3.13	16.33 ± 3.16	<0.001	0.28
Leg press 10 RM (kg)	77.0 ± 4.14	88.75 ± 4.13	<0.001	0.81	79.55 ± 6.62	89.77 ± 4.94	<0.001	0.65
Knee extension 10 RM (kg)	39.0 ± 5.01	43.0 ± 5.01	<0.001	0.37	41.11 ± 8.43	44.88 ± 8.31	<0.001	0.21
Triceps skinfold (mm)	9.33 ± 0.55	8.85 ± 0.56	<0.001	0.39	9.44 ± 0.67	8.94 ± 0.66	<0.001	0.35
Biceps skinfold (mm)	8.08 ± 0.60	7.55 ± 0.70	<0.001	0.37	8.01 ± 0.62	7.55 ± 0.64	<0.001	0.34
Flexed arm circumference (cm)	28.61 ± 0.49	29.03 ± 0.45	<0.001	0.40	28.47 ± 0.52	28.92 ± 0.54	<0.001	0.39

Legends: MJ—multi-joint group; MJ+SJ—multi and single-joint group; ES—effect size.

**Table 4:** Change in outcomes over the training period (mean ± standard deviation) in addition to 95%CIs.

	MJ Group		MJ+SJ Group		ANCOVA	
	Change	95% CIs	Change	95% CIs	*F*	*p*
Bench press 10 RM (kg)	3.1 ± 0.3	2.5 to 3.8	2.4 ± 0.3	1.8 to 3.1	2.465	0.139
Triceps 10 RM (kg)	2.1 ± 0.2	1.6 to 2.6	2.4 ± 0.2	2.0 to 2.9	0.945	0.348
Pulldown 10 RM (kg)	2.4 ± 0.2	2.0 to 2.8	2.0 ± 0.2	1.6 to 2.4	2.563	0.132
Biceps 10 RM (kg)	2.0 ± 0.1	1.8 to 2.2	1.9 ± 0.1	1.7 to 2.1	0.837	0.376
Leg press 10 RM (kg)	11.4 ± 0.7	9.9 to 12.9	10.5 ± 0.7	9.1 to 12.0	0.758	0.399
Knee extension 10 RM (kg)	4.0 ± 0.2	3.6 to 4.4	3.8 ± 0.2	3.4 to 4.2	0.638	0.438
Triceps skinfold (mm)	−0.49 ± 0.03	−0.55 to −0.43	−0.50 ± 0.03	−0.56 to −0.44	0.091	0.768
Biceps skinfold (mm)	−0.54 ± 0.04	−0.63 to −0.46	−0.45 ± 0.04	−0.53 to −0.37	2.545	0.133
Flexed arm circumference (cm)	0.43 ± 0.03	0.36 to 0.49	0.45 ± 0.03	0.40 to 0.51	0.434	0.521

Legends: MJ—multi-joint group; MJ+SJ—multi and single-joint group.
