# Expression of Concern: Barbalho et al. Effects of Adding Single Joint Exercises to a Resistance Training Programme in Trained Women. *Sports* 2018, *6*, 160

**DOI:** 10.3390/sports14060240

**Published:** 2026-06-10

**Authors:** Sports Editorial Office

**Affiliations:** MDPI, St. Alban-Anlage 66, 4052 Basel, Switzerland; sports@mdpi.com

With this notice, the *Sports* Editorial Office and Editorial Board wish to alert readers to concerns related to this article [1]. Following publication, concerns were raised regarding the validity of the data underlying this article.

The authors of this publication and the appropriate institution have been contacted for further clarification. The investigation is being coordinated by the Editorial Office with the involvement of the Editorial Board. Once this process is complete, the Editorial Office will provide an update on this situation and announce any post-publication modification deemed to be necessary, as per MDPI’s Updating Published Papers policy (https://www.mdpi.com/ethics#_bookmark30, accessed on 8 June 2026).
